# Moderate Beer Intake and Cardiovascular Health in Overweight Individuals

**DOI:** 10.3390/nu10091237

**Published:** 2018-09-05

**Authors:** Teresa Padro, Natàlia Muñoz-García, Gemma Vilahur, Patricia Chagas, Alba Deyà, Rosa Maria Antonijoan, Lina Badimon

**Affiliations:** 1 Cardiovascular ICCC-Program, Research Institute Hospital de la Santa Creu i Sant Pau, IIB-Sant Pau, 08025 Barcelona, Spain; tpadro@santpau.cat (T.P.); nmunoz@santpau.cat (N.M.-G.); gvilahur@santpau.cat (G.V.); patriciachagas.ufsm@hotmail.com (P.C.); albadeya4@gmail.com (A.D.); 2 CIBERCV Instituto de Salud Carlos III, 28029 Madrid, Spain; 3 Department of Food and Nutrition, Universidade Federal de Santa Maria, Palmeira das Missões RS 98300000, Brasil; 4 Medicament Research Center (CIM), Research Institute Hospital de la Santa Creu i Sant Pau, IIB-Sant Pau, 08025 Barcelona, Spain; rantonijoana@santpau.cat; 5 Cardiovascular Research Chair, UAB, 08025 Barcelona, Spain

**Keywords:** cardiovascular-risk-factors, overweight, obesity, fermented-beverage, lipoprotein-oxidation, HDL-antioxidant-capacity, cholesterol-efflux, endothelial-function

## Abstract

Consistent epidemiological evidence indicates that low-to-moderate alcohol consumption is inversely associated with cardiovascular event presentation, while high levels of alcohol intake are associated to increased cardiovascular risk. Little is known on the effects of moderate beer intake in the metabolic syndrome. The aim of this study is to investigate the effects of moderate and regular daily intake of beer with meals in overweight (body mass index (BMI) of 28–29.9 kg/m^2^) or obese class 1 (BMI of 30–35 kg/m^2^) individuals without other cardiovascular risk factors (dyslipidemia, type 2-diabetes, hypertension) focusing on the effects related to changes in weight, in lipoproteins and vascular endothelial function. We have performed an open, prospective two-arms longitudinal crossover study to investigate the effects associated with regular consumption (four week) of alcohol-free-beer (0 g alcohol/day) or traditional-beer (30 g alcohol/day in men and 15 g alcohol/day in women) on anthropometrical and biochemical parameters, liver and kidney function biomarkers, and vascular endothelial function. After four-week intervention with traditional and/or alcohol-free beer, BMI did not show any significant change and values for liver and kidney functions were within the normal levels. Moderate traditional beer intake did not affect lipid levels—however it significantly increased the antioxidant capacity of high density lipoprotein (HDL). In addition, apoB-depleted serum (after the four-week intervention period) showed a higher potential to promote cholesterol efflux from macrophages. Beer consumption did not induce vascular endothelial dysfunction or stiffness. In summary, our results based on a 12-week prospective study provide evidence that moderate intake of beer (traditional and alcohol-free) does not exert vascular detrimental effects nor increases body weight in obese healthy individuals. In contrast, moderate intake of beer increases the anti-oxidative properties of HDL and facilitates cholesterol efflux, which may prevent lipid deposition in the vessel wall.

## 1. Introduction

Epidemiological studies have reported a J-shaped relationship between alcohol intake and cardiovascular disease (CVD). Therefore, low-to-moderate drinkers have a lower risk of developing coronary heart disease and less mortality compared to both heavy drinkers and abstainers, being the heavy drinkers the ones with the highest risk [1,2,3]. However, the benefits of alcohol consumption are widely discussed and remain controversial regarding the type of beverage [4,5,6] and drinking pattern [7,8]. Mukamal et al. reported that alcohol intake distributed over the week inversely associates with the risk of myocardial infarction, independently of the type of beverage or the proportion consumed with meals [7]. In contrast, results of the INTERHART Study, a case-control study examining the relationship between alcohol consumption and the long- and short-term risk of myocardial infarction (MI) in all inhabited continents of the world, highlights the importance of the type of alcohol consumed and the pattern of alcohol use as modifiers of the relationship between alcohol and myocardial infarction [8]. In agreement, some studies indicate that low-to-moderate alcohol consumption does not have a net mortality benefit compared to abstainers because the rates of mortality and cardiovascular disease (CVD) risk from alcohol are significantly altered by study design and characteristics as well as confounding factors [9,10,11,12]. Regarding the type of beverage, early studies supported the benefits of wine on cardiovascular outcomes and mortality and depicted that a J-shaped relationship was found in wine, but neither in beer nor spirits [13,14]. However, more recently, an updated meta-analysis study reported by Costanzo et al. [15] provided evidence that the J-shaped association is found in both wine and beer, but not in spirits. Fermented beverages, both wine and beer, are rich in antioxidants, mainly polyphenolic compounds [5,16,17], that are missing in spirit beverages.

Beer is one of the most consumed alcoholic beverages in the world; in America it is the most popular alcoholic beverage, contributing up to 55.3% of the alcohol consumed [18]. Studies on traditional- and non-alcoholic beer consumption are needed to evidence their effects in different factors contributing to cardiovascular health and their benefit/risk ratios. Here, we are reporting a clinical study aimed to investigate beneficial and detrimental effects of moderate and regular intake of beer in overweight or obese class-1 individuals without other cardiovascular risk factors (CVRF) such as dyslipidemia, type 2-diabetes or hypertension, focusing on the effects related to changes in weight, on lipoprotein atheroprotective effects and vascular endothelial function.

## 2. Materials and Methods

### 2.1. Subjects

Healthy adult men and women between ages of 40–60 years (*N* = 36), non-smokers, regular but moderate beer consumers (self-reported alcohol consumption), and with overweight (body mass index (BMI) of 28–29.9 kg/m^2^) or obesity class 1 (BMI of 30–35 kg/m^2^) were invited to participate in the study through word of mouth and a newspaper advertisement. Moderate beer drinking was defined according to the “Dietary Guidelines for Americans 2015–2020,” U.S. Department of Health and Human Services and U.S. Department of Agriculture, (https://www.niaaa.nih.gov/alcohol-health/overview-alcohol-consumption/moderate-binge-drinking) and refers up to 1 drink per day for women, and up to 2 drinks per day for men. Subjects were excluded if they reported extensive consumption of beer (>60 g day of ethanol), existing chronic illnesses including cancer, overt hyperlipidemia, diabetes mellitus, hypertension, heart, liver or kidney disease. Other exclusion criteria included the use of lipid-lowering drugs, beta-blockers or diuretics, history of CVD, psychiatric illness or treatment of psychotropic drugs, intolerance to alcoholic beverages or being in a weight-loss program. To confirm health status, all subjects underwent a complete physical examination conducted by the study physician before entry into the study. The study complies with the Declaration of Helsinki and was approved by the Human Ethical Review Committee of the Hospital “Santa Creu i Sant Pau” of Barcelona (Ref 14/186; 12 November 2014). Informed written consent was obtained from all participants before entering the study. 

### 2.2. Study Design and Dietary Monitoring

**Figure 1:**
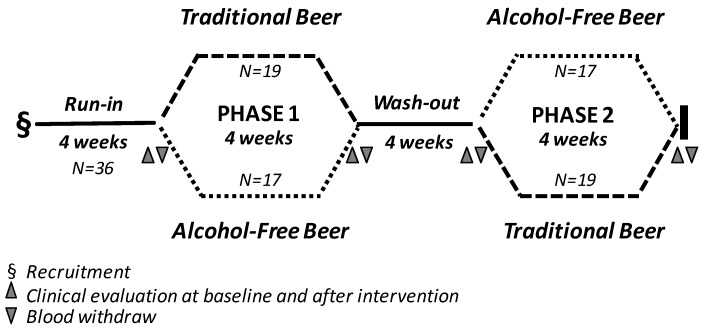
Flow diagram describing the study design.

The study was an open, randomized two-arm longitudinal cross-over trial with a 4 week intervention period (Figure 1). All subjects were subjected to two 4-week treatment sequences, separated by a 4-week wash-out period. Before the initiation of the intervention, individuals were subjected to a 4-week run-in period. At the end of the run-in period, subjects were randomly allocated to receive one of the two treatment sequences (study arm-1: traditional beer in the first intervention period and alcohol-free beer in the second intervention period; study-arm-2: non-alcoholic beer in the first intervention period and traditional beer in the second intervention period). During the intervention periods, men and women were instructed to drink two cans (660 mL beer) and one can (330 mL beer), respectively, of traditional beer (15 g of ethanol and 604 mg polyphenols/can) or alcohol-free beer (0.0 g alcohol and 414 mg polyphenols/can) per day. During the run-in and wash-out periods and throughout the intervention phases the participants were asked to maintain their physical activity level and usual dietary habits, abstaining from drinking alcoholic beverages and alcohol-free beer out of those provided as part of the study. Dietary habits, determined by using food frequency questionnaires, were recorded prior to each visit, and rare changes in diet habits were reported.

Compliance was monitored by regular telephone contact with participants and interviewing them at the end of each intervention period. Participants also recorded whether they had consumed beer on a diary card each day. Moreover, at the end of each intervention period, a clinician assessed any side effects or symptoms such as flushing, bloating, dizziness, vomiting, diarrhea, with possible association with the study interventions.

Traditional and alcohol-free beers were of the lager type from the same Spanish commercial brand. The phenolic composition derived from the traditional and alcohol-free beer interventions and the daily intake according to the gender is provided in Supplementary Table S1.

### 2.3. Blood Samples

Twelve hour fasting blood samples were collected on days 1 and 28 (baseline and endpoint of the first treatment period) and on days 56 and 84 (baseline and endpoint of the second treatment period). Blood samples were collected without anticoagulant or in ethylenediamine tetraacetic acid (EDTA)-containing Vacutainer tubes for serum and plasma preparation, respectively. Serum and plasma fractions were separated by centrifugation at 1800× *g* for 20 min and stored at −80 °C until used.

### 2.4. Anthropometric Data, Blood Pressure, Serum Lipid Profile and Other Biochemical Measurements

Anthropometric measurements were determined at baseline, before starting the intervention, and at the end of the intervention periods (see Figure 1). The BMI was calculated using the formula body weight (Kg)/height (m^2^) [19]. Waist circumference (WC) was measured between the lowest rib and the iliac crest with the participant standing.

Serum biochemical measurements were performed using routine commercially available assays for glucose levels, hepatic and renal markers, hemogram and standard serum lipid profile including triglycerides, total cholesterol (TC) and HDL (high density lipoprotein) cholesterol (Roche Diagnostics, Basel, Switzerland). As there were no cases of hypertriglyceridemia, LDL (low density lipoprotein) cholesterol was calculated using the Friedewald equation. Glomerular filtration rate was obtained according to the CKD-EPI Levey equation [20].

### 2.5. LDL and HDL Sample Preparation, Purity Control and Oxidation Assays

#### 2.5.1. Lipoprotein Preparation

LDL (density range 1.019–1.063 g/mL) and HDL (density range 1.063–1.210 g/mL) were obtained from plasma-EDTA from individual samples by sequential ultracentrifugation, according to the method originally described by Havel et al. [21] and modified by De Juan-Franco et al. [22]. Briefly, plasma was adjusted to a density of 1.019 g/mL with a concentrated salt solution (potassium bromide) and centrifuged at 225,000× *g* (18 h) in a Beckman L-60 ultracentrifuge with a fixed-angle type 50.4 Ti rotor (Beckman, Brea, CA, USA). After removal of the top layer, containing very low and intermediate density lipoproteins (VLDL and IDL), the density of the infranatant was adjusted to 1.063 g/mL, followed by centrifugation for 20 h at 225,000× *g* and LDL were collected from the top of the tube. Lastly, the process was repeated adjusting plasma density to 1.210 g/mL and samples ultracentrifuges at 225,000× *g* for 24 h, at 4 °C, to allow HDL to float and separate from lipoprotein deficient serum.

In addition, LDLs to be used in the TRAP assay were isolated from a pool of plasma obtained from normolipemic subjects and obtained as described above in a Beckman Optima L-100 XP with a fixed- angle type 50.2 Ti (Beckman, Brea, CA, USA).

LDL and HDL fractions were dialyzed against phosphate buffer saline 1X (PBS 1X) for 24 h. LDL- and HDL-protein content was determined by the colorimetric assay BCA (Pierce, Thermo Fischer Scientific, Waltham, MA, USA), and adjusted to 100 μg/mL. Samples were left protected of light at 4 °C until analysis. LDL and HDL purity was routinely analyzed by electrophoresis in agarose gels (SAS-MX Lipo 10 kit, Helena Biosciences, Gateshead, UK).

#### 2.5.2. Conjugated Dienes Assay

Susceptibility of LDL to copper-induced oxidation was assessed by determining the formation of conjugated dienes. Briefly, freshly prepared LDL samples adjusted to 100 μg/mL with PBS 1X were analyzed by incubation with a copper (II) sulfate (CuSO_4_·5H_2_O) solution at a final concentration of 5 μM. The change of absorbance was determined during 2.5 h at 37 °C using a SpectraMax 190 Microplate reader (Molecular Devices, Philadelphia, PA, USA) by continuously monitoring the formation of conjugated dienes, a product of lipid peroxidation with absorbance peak at 234 nm. The total amount of conjugated dienes was calculated as previously described [23].

#### 2.5.3. HDL Antioxidant Potential

The antioxidant potential of HDL was assessed by performing the total radical-trapping antioxidative potential (TRAP) test [24], a method based on the capability of HDL to prevent LDL oxidation. Briefly, HDL and LDL lipoproteins were diluted in PBS 1X to a final concentration of 100 µg protein/mL. LDL derived from the control plasma pool were incubated with CuSO_4_·5H_2_O (final concentration of 20 µM) in the absence/presence of HDL from each individual subject for 4 h (37 °C). Afterward, oxidation was stopped with 1 mM EDTA and samples incubated with 10 µM DCFH-DA (2′,7′-dichlorodihydrofluorescein diacetate) for detection of the oxidation level [25]. Intensity of fluorescence was determined with a Typhoon FLA9500 (GE Healthcare, Chicago, IL, USA) set at λex = 500 nm and λem = 520 nm. Final fluorescence measurements were expressed as the percentage of oxidized LDL generated in the presence of HDL relative to the oxidation level when LDLs were incubated in the absence of HDL.

### 2.6. HDL Cholesterol Efflux Capacity Assay 

The cholesterol efflux capacity of HDL was determined in cholesterol-loaded murine macrophages as previously reported [26,27]. To this end, J774A.1 mouse macrophages were cultured in RPMI 1640 (Roswell Park Memorial Institute medium) containing 10% of heat-inactivated FBS (Fetal bovine serum), 2 mM glutamine, 100 U/mL penicillin, 100 U/mL streptomycin and 10 μg/mL gentamicin at 37 °C in a humidified atmosphere of 5% CO_2_.

For the experiments, macrophages (1.5 × 10^5^ cells/well seeded in 6-well culture plates) were labeled for 48 h with [1α, 2α (n)^−3^H cholesterol] (GE Healthcare, Chicago, IL, USA) at 1 μCi per well. Cells were equilibrated overnight in 0.2% bovine serum albumin (BSA) and thereafter incubated with RPMI media containing 5% apoB-depleted serum (4 h, 37 °C) to promote cholesterol efflux from the [^3^H] cholesterol-labeled cells. Radioactivity signal was quantified in both media and cells and the percentages of cholesterol efflux calculated by expressing the radioactive cholesterol released to the medium as the fraction (%) of the total radioactive cholesterol present in the well (radioactivity in the cell + radioactivity in medium).

### 2.7. Vascular Endothelial Function and Arterial Stiffness

Endothelial function and arterial stiffness were assessed by digital plethysmography using the EndoPAT2000-device (Itamar Medical Ltd., Caesarea, Israel). Measurements were performed according to the manufacturer’s instructions in subjects resting in supine position and both hands on the same level in a comfortable, thermoneutral environment, with a temperature of 21–24 °C. Arterial systolic and diastolic blood pressure and heart rate frequency were measured before starting the test. Pneumatic probes were placed on each index finger and a blood pressure cuff on one arm (study arm), while the contralateral arm served as a control (control arm). After a 10-min equilibration period, the blood pressure cuff on the study arm was inflated to 60 mmHg above systolic pressure for 5 min. The cuff was then deflated to induce reactive hyperemia (RH), whereas the signals from both PAT channels (Probe 1 and Probe 2) were recorded by a computer.

The recording was initiated after 25 min of rest. After 5 min baseline recording, the blood pressure cuff was inflated to 60 mmHg above systolic blood pressure and no less than 200 mmHg. Occlusion was confirmed by visual confirmation of complete attenuation of the PAT signal from the test arm. After 5 min occlusion, the cuff was deflated, and the recording continued for 5 min during the reactive hyperemia phase. Recordings from the non-occluded arm served as an internal control correcting for systemic changes in vascular tone. Endothelial function and arterial stiffness were calculated using the EndoPAT software package version 3.4.4. Endothelial function was given as the reactive hyperemia index (RHI) and the arterial stiffness as the augmentation index (AI) and AI standardized to a pulse of 75/min (AI@75) [28].

### 2.8. Inflammatory Markers

Plasma levels of CRP (C-reactive protein), TNF-α (Tumour Necrosis Factor-alpha), and IL-6 (Interleukin-6) were measured by an Enzyme-Linked ImmunoSorbent Assay (ELISA): High sensitivity CRP and TNF-α (Quantikine HS ELISA, R&D Systems, Minneapolis, MN, USA), IL-6: (AssayMax, AssaPro, St. Lake Charles, LA, USA). All assays were performed according to the manufacturer instructions.

### 2.9. Statistical Analysis

Statistical analyses were conducted using StatView 5.0.1 software (SAS Institute, Cary, NC, USA) and SPSS software (IBM SPSS Statistics 25.0.0, New York, NY, USA) except when indicated. Data are expressed by the number of cases (qualitative variable) and as mean ± SEM or median [IQR] for the quantitative variable. For all analyzed variables, values at the end of the run-in and the wash-out period were considered as the baseline value for the following intervention period. Differences in the baseline characteristics of the groups and in the percentage of change between intervention-diets were analyzed by unpaired Student’s *t*-test for parametric variables and chi-square test for non-parametric variables. Effects of the 4-week interventions were evaluated using a paired Student’s *t*-test (baseline and post-intervention values) or an analysis of variance (ANOVA) test introducing the different obesity and lipid-related variables as co-variable when required. All reported *p*-values are two-sided, and a *p*-value of 0.05 or less was considered to indicate statistical significance.

## 3. Results

### 3.1. Baseline Characteristics of the Study Population and Side Effects of Beer Consumption

Thirty-six subjects (21 men, 15 women) with an average age of 48.3 ± 5.4 years who were initially recruited for the study completed both intervention phases and were included in the final analysis. Table 1 shows the characteristics of study population at baseline, after the run-in period (Phase 1) and after the wash-out period (Phase 2), at the time of starting each intervention period. All subjects included in the study had overweight (BMI values in the range 28.0–29.9 kg/m^2^, *N* = 18) or obesity class-1 (BMI values in the range 30.0–35.0 kg/m^2^, *N* = 18). BMI was similar between men and women, being mean BMI 30.2 ± 0.4 kg/m^2^ in men and 30.6 ± 0.7 kg/m^2^ in women (Student’s *t*-test, *p* = 0.554).

As shown in Table 2, after a four-week intervention with traditional or alcohol-free beer, anthropometric variables such as body-weight, BMI, and waist circumference, the hemodynamic parameters, plasma levels of hepatic and kidney function, and hemogram profile remain within the normal physiological range. A modest increase (within the normal-range) in glucose and GGT levels was found after four-week intervention with traditional beer (*p* < 0.05). 

Similar results were found when women and men (42% and 58% respectively of the study population) were analyzed separately. Therefore, despite small differences were detected relative to baseline for cardiac frequency, aspartate transaminase (AST) levels, creatinine levels and estimated glomerular filtration, all changes remained within the normal physiological range in the men-subgroup (Supplementary Table S2). Changes relative to baseline induced by intervention with traditional or alcohol-free beer did not differ in the woman-subgroup (Supplementary Table S3). The fact that the changes were within normal range indicate that there was no toxicity associated to beer uptake in a highly compliant group.

### 3.2. Effects of Beer Consumption on Weight Indexes

Neither traditional nor alcohol-free beer induced any weight gain in the study population.

Table 3 shows evolution of weight, BMI, and waist circumference for men and women during the study period (12-week).

### 3.3. Effect of Beer Consumption on Lipid Profile and Lipoprotein Functionality

#### 3.3.1. Lipid Profile

Mean serum lipid concentration at baseline and after of each intervention period are presented in Table 4. Mean serum concentrations of TC, non-HDLc, LDLc, HDLc, VLDLc and TG did not show any significant change after intervention with traditional beer or alcohol-free beer, when the total population was considered. Interestingly, levels of HDLc and TC were significantly increased after intervention with traditional beer in the subgroup of subjects with LDLc levels < 130 mg/dL (moderate CVD risk), whereas this effect was not found in subjects with LDLc > 130 mg/dL (Table 5).

#### 3.3.2. LDL Susceptibility to Oxidation

The susceptibility of LDL to oxidation was assessed by the maximal amount of generated conjugated dienes (Dmax) and the maximum velocity of dienes production (Vmax), during in vitro incubation of purified LDL with cupric ions. Susceptibility of LDL to oxidation (Dmax and Vmax) did not relate to the baseline BMI, and did not differ significantly between men and women (data not shown).

**Figure 2:**
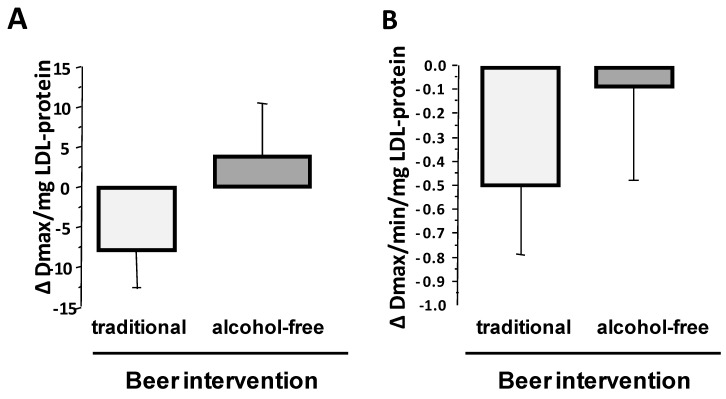
Effect of four-week intervention with alcohol-free and traditional beer on the susceptibility of plasma LDL to be oxidized. Values are given as change verses baseline for (**A**) maximal value for generated conjugate dienes, and (**B**) Vmax that conjugate dienes are generated. Bars refer to mean values and lines to standard error of the mean (SEM).

Sixty-one percent of the participants (52% of men and 73% of women) showed lower susceptibility of LDLs to oxidation after four-week intervention with traditional beer as compared to control baseline LDLs, while around 42% of participants (43% of men and 40% of women) showed less susceptibility of LDLs to oxidation after alcohol-free beer intake (*p* = 0.007 for the difference in the response to both interventions by the X^2^-test). Compared to baseline, mean level of Dmax was slightly lower (−8.0 ± 4.8 Dmax/mg LDL-protein) after intervention with traditional beer (*p* = 0.052; Figure 2A) and a similar trend was found for the kinetic of conjugated dienes (CD) generation (Decrease in Vmax: *p* = 0.075; Figure 2B), but changes were non-significant.

#### 3.3.3. HDL Antioxidant Capacity

**Figure 3:**
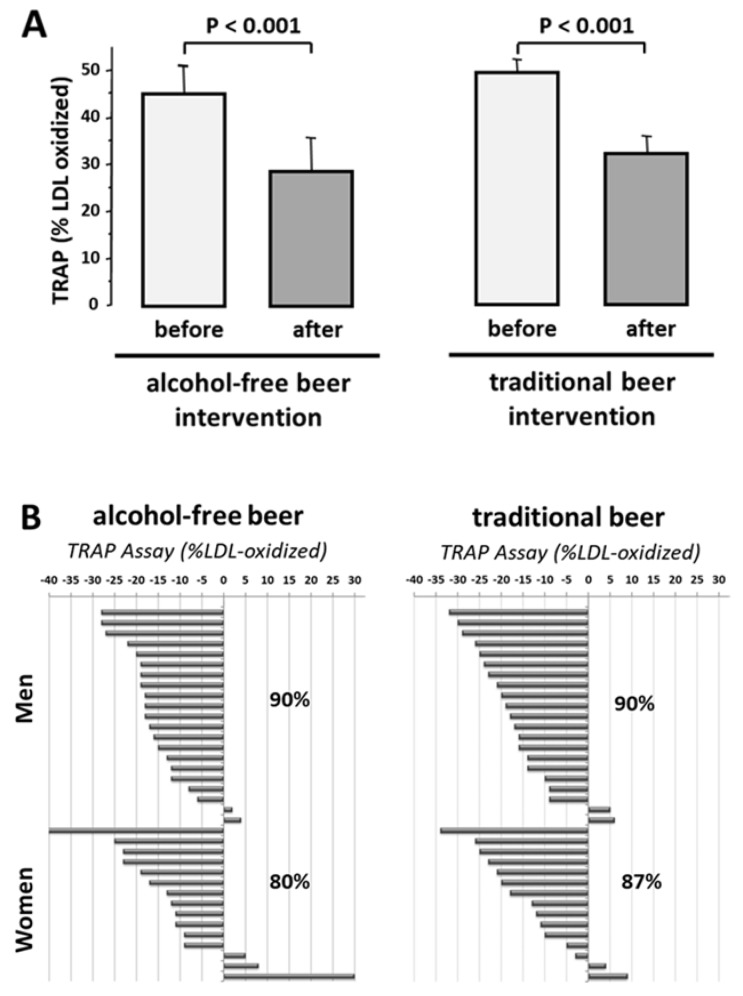
Effect of moderate beer consumption on HDL antioxidant capacity in subjects with overweight or obesity class-1. (**A**) Results are expressed as % of oxidized LDL referred to the value obtained in the absence of HDL. (**B**) Functional response of HDL to oxidative stress at individual level.

Susceptibility of LDL to oxidation (Dmax) in the presence of HDL was decreased to 47.3 ± 1.8% of the value obtained in the absence of high-density lipoproteins (*p* < 0.001). This value was unrelated to sex (men: 47.4 ± 2.1%; women 47.3 ± 3.51%). After four-week intake of both types of beer, HDL exhibited higher antioxidant effects than control baseline HDL (*p* < 0.001, Figure 3A). These beneficial effects on HDL of beer (5–40% decrease in LDL oxidation compared to the effect induced by HDL obtained at baseline) was found in 87% of the subjects after intervention with traditional beer and 80% after intake of alcohol-free beer (Figure 3B). This effect did not relate to sex (men: Traditional-beer, −17.2 ± 2.2%; alcohol-free beer: −15.7 ± 1.8%); women: Traditional-beer: −13.8 ± 3.0%; alcohol-free beer: −11.3 ± 4.2%).

#### 3.3.4. Effect of Beer Intake on HDL Cholesterol Efflux Capacity

After intervention with traditional beer, there was a statistically significant increase in the capacity of apoB-depleted serum to induce cholesterol efflux from macrophages in vitro (*p* = 0.002; Table 6). Intervention with alcohol-free beer did not induce any statistically significant change on cholesterol efflux mediated by the apoB-depleted serum.

### 3.4. Plasma Inflammatory Markers and Beer Intervention

Regular intake of traditional or alcohol-free beer did not induce any increase in systemic inflammatory markers such as CRP, IL6, TNF alpha (Table 7).

### 3.5. Vascular Endothelial Function and Arterial Stiffness before and after Intervention

Mean value of vascular reactivity measured by the pulse amplitude response to hyperemia (response hyperemia index; RHI), in subjects with overweight or obesity was 1.69 ± 0.43. This value indicates normal endothelial function, because RHI values below 1.67 are categorized as endothelial dysfunction, whereas higher RHI values are considered normal function.

Four-week intervention with traditional or alcohol-free beer did not induce any statistically significant effect on the RHI level compared with baseline when the total study population was considered (before vs. after intervention: 1.71 ± 0.05 vs. 1.75 ± 0.07 and 1.67 ± 0.05 vs. 1.64 ± 0.05, for traditional and alcohol–free beer, respectively). Interestingly, 24 subjects that showed endothelial dysfunction at entry (baseline RHI values: 1.38 ± 0.03) had a statistically significant improvement in endothelial function after non-alcoholic beer intake, while those with values within the normal RHI range at entry (2.02 ± 0.11, *N* = 12) did not (RHI change: +0.092 ± 0.04 vs. −0.381 ± 0.11, *p* = 0.001). Similar trend was found after intervention with traditional beer, although differences did not achieve statistical significance (change in RHI group < 1.67 vs. RHI-group > 1.67: +0.137 ± 0.07 vs. −0.075 ± 0.13, *p* = 0.113). Indeed, changes in RHI value after beer intervention negatively correlated with RHI values at baseline (*r* = −0.567, *p* < 0.001). Therefore, beer intake did not induce any impairment in endothelial vascular reactivity and function.

The augmentation index (AI) was calculated based on the EndoPAT data and is considered a marker for the arterial stiffness. AI standardized to a pulse of 75/min (AI@75) gave baseline values of −5.61 ± 1.81 in men and +13.46 ± 3.62 in women (*p* < 0.001). Four-week intervention with traditional beer or with non-alcoholic beer did not lead to impairment in AI@75 values (traditional beer: +2.26 ± 1.43 verses baseline, *p* = 0.229; non-alcoholic beer: −1.23 ± 1.00 verses baseline, *p* = 0.123). Beer intake did not induce arterial stiffness.

### 3.6. Beer Consumption and Cardiovascular Risk Score

The risk of CVD in the study population was calculated on basis to the Framingham risk score (FRS), which gave a mean value of 7.8 ± 0.5, being the risk 8.7 ± 1.1 in women and 7.3 ± 0.5 in men. As shown in Table 8, the FRS was not increased after daily intake of alcohol-free or traditional beer during a total eight-week period. These values refer to a 10-year CV-risk of 3% in men and <1% in women at baseline. These percentages did not change after regular consumption of alcohol-free or traditional beer during four-week periods.

## 4. Discussion

Although excessive alcohol consumption is unquestionably a health hazard, there is substantial evidence based on epidemiological and observational studies suggesting that a light-to-moderate regular alcohol intake has a protective effect in overall mortality, mainly from coronary artery disease [30]. This evidence is supported by data derived from experimental animal studies and prospective clinical trials suggesting that alcoholic beverages may exert different protective effects against atherosclerosis development either by modulating lipid metabolism, platelet activity, inflammation, and thrombogenic factors [31,32]. Despite the results of these studies, findings up to now are inconclusive. The balance between risks and benefits of moderate alcohol consumption in health is still under discussion [33,34]. Alcohol is the main active component in alcoholic beverages and as such considered as the causal factor in both beneficial and toxic effects. However, nowadays particular interest focuses on fermented alcoholic beverages such as wine or beer. In this respect, epidemiological evidence [17] and results from prospective clinical trials [5] suggests that these beverages with heterogenous content of non-alcoholic components might confer better cardiovascular protection than spirits.

In this study we performed a randomized, cross-over, prospective study to investigate benefits and risks of moderate intake of beer in low cardiovascular risk individuals with overweight or obesity class1 (BMI 27–35 kg/m^2^). More specifically, effects related to changes in weight, on lipoprotein atheroprotective effects, and vascular endothelial function, were investigated. Our results provide consistent evidence that regular consumption of alcohol-free beer or traditional beer in moderate quantities (two cans a day for men and one can a day for women) over two periods of four weeks did not modify or only induced minimal changes within clinical normality range in plasma biomarkers of liver and kidney function, whereas significantly promoted atheroprotective properties of HDL, such as prevention of LDL oxidation and induction of cholesterol efflux from macrophages, considered a first step in the reverse cholesterol transport [35]. Supporting the findings of our study, Romeo et al. did not report any change regarding hepatic enzymes in younger healthy men and women of lesser BMI (24–25 kg/m^2^) after a four-week alcohol abstinence and a four-week moderate consumption of a Pils-style beer [36].

One of the most important questions regarding moderate beer consumption is whether it induces an increase in body weight and waist circumference, since these anthropometric parameters are associated to the increase in cardiovascular risk [37] atherosclerosis [38] and also mortality [39,40]. Here, the moderate consumption of traditional or non-alcoholic beer for a total of eight weeks did not induce significant changes in body weight, BMI or waist circumference in our study population of overweight/obese, but otherwise healthy subjects. Interestingly, Chiva-Blanch et al. reported that regular intake of traditional or non-alcoholic beer did not alter the weight, BMI, or waist-hip ratio in men at high cardiovascular risk, because of previous clinical evidence of disease or presence of CVD risk factors [5].

Excessive alcohol intake has been associated with hypertension and atrial fibrillation [41]. In our study, moderate beer intake during 4 + 4 week-periods did not affect blood pressure or heart-rate. Moreover, moderate intake of regular beer did not modify glucose levels after a four week intervention in our adult population with baseline values below 90 mg/dL, neither affected plasma levels of liver enzymes nor biomarkers for kidney dysfunction beyond the normal range. It is to notice that four-week regular beer intake increased in 15% the plasma GGT levels, although the increase was within the standards considered normal. This finding can be justified due the fact that the alcohol is primordially metabolized by the enzyme alcohol dehydrogenase (ADH) in the hepatocytes [42] and therefore liver is a highly sensitive organ for detecting alcohol-induced changes [43,44,45].

Thus, the above results provided evidence that regular (daily) but moderate intake of beer during an eight-week intervention study does not induce harmful effects on the hepatic or renal function, neither affects body weight, plasma glucose or blood pressure pattern beyond the normal range in healthy subjects in spite of presenting with overweight or obesity. According to these findings, light-to-moderate beer consumption (women ≈ 15 g/day alcohol, men ≈ 30 g/day alcohol) does not show any detrimental effects and in contrast it might improve the atheroprotective profile of their HDL. However, further studies in larger and more heterogeneous populations with longer duration of beer consumption periods may be necessary to prove the effects of moderate beer intake on HDL function and their effect on the vessel wall and on CVD prevention.

A key element during atherosclerosis progression is the accumulation of cholesterol within macrophages in the arterial wall and their transformation in foam cells with a more atherogenic phenotype. In this respect, HDL have a relevant atheroprotective role by promoting the reverse cholesterol transport (RCT) from peripheral tissue (i.e., macrophages on the arterial intima) with the subsequent excretion of cholesterol out of the body after transport to the liver [46,47]. HDL has the capacity to remove cholesterol from cells acting as acceptor during the cholesterol efflux, which is the first step of the RCT [35,48]. By using apoB-depleted serum to measure of HDL function [49], we provided evidence that moderate but regular intake of traditional beer favors HDL-induced cholesterol efflux. It had previously been reported that alcohol consumption at the dose of 40 g alcohol/day for 17 to 23 days increased ABCA1-mediated cholesterol efflux [48]. Here, we have seen that the beneficial effect of beer intake on HDL efflux capacity is already evident after a low-moderate alcohol intake (15 g alcohol/day in women and 30 g alcohol/day in men) [50].

Using a preclinical swine model of dyslipidemia, we had shown that the intake of both alcohol and non-alcohol beer reduces the systemic oxidative stress triggered by hypercholesterolemia through a process mediated by HDL [51]. The current study in humans further supports the concept of a functional effect of beer intake, indistinctly of its alcohol content, enhancing the antioxidant capacity of HDL. Epidemiological and observational studies have associated the beneficial effects of alcohol in cardiovascular protection to their effect on plasma HDL cholesterol levels [52,53]. In this regard, results of a recently reported community-based study with more than 70.000 subjects over six years suggest that moderate alcohol consumption associates with less HDL-cholesterol decrease over time [6]. In our study, four-week regular intake of moderate amount of traditional beer only raised HDL levels in subjects with a low LDL-lipid profile (<130 mg/dL). However, the four-week term was already able to improve circulating HDL quality by rendering HDL particles functionally active to prevent LDL from oxidation and facilitate cell-cholesterol efflux.

Impaired endothelial function is the first step in the process of atherosclerosis, usually induced by dyslipidemia, even before the development of the fatty streak [54,55]. In our study there was no impairment in endothelial function due to beer intake in overweight/obese individuals with normal LDL levels. Previous studies have shown that regular beer-intake protects against hypercholesterolemia-induced coronary endothelial dysfunction as we have demonstrated in the hypercholesterolemic swine model [51]. Moreover, a beneficial effect on vascular endothelial function was reported for acute beer intake [56]. Similarly, beneficial acute beer intake effects were reported on arterial function and structure [57,58] in healthy young lean subjects. So far, to our knowledge, there are not data regarding a long-term effect of beer on vascular dysfunction and atherosclerosis related arterial biomarkers, such as flow mediated dilatation (FMD) and augmentation index (AI). Here, assessment of the endothelial function (EndoPAT technique) at baseline and after four-week beer consumption did not induce any change in endothelial vascular reactivity and function. Indeed, the reported beneficial effects of beer on endothelial function after acute beer intake were lost four hours after the intake of the alcoholic beverage [56], suggesting that beer intake may induce beneficial acute short-term changes on the vascular endothelium.

The current study has some limitations that warrant discussion. Although this study is based on a well-characterized cohort of healthy but overweight/obese individuals, it has a small sample size that does not represent a more general population of individuals with metabolic syndrome. Moreover, due to the limited number of women in the study, we cannot exclude that some sex related effects could result from a small but differential response in the peri-menopausal women group (women median [IQR] age: 50 (44–54) years) of our study and might not reflect the effects of beer intake in women in general. However, the longitudinal cross-over design gives strength to the results of the study minimizing inter-individual variability for the effects observed with the traditional and alcohol-free beer. A second aspect is the short duration of the intervention for each type of beer. Although many studies that focus in variables like those analyzed here use these same intervention periods, our results might not reflect the potential risks/benefits of longer-term moderate beer consumption.

## 5. Conclusions

In summary, moderate intake of beer (traditional and alcohol-free) does not exert vascular detrimental effects nor increases body weight in obese but otherwise healthy individuals during the eight-week intervention study. In contrast, moderate intake of beer was associated with favorable effects on HDL-function increasing its capacity to protect against LDL oxidation and to enhance cholesterol efflux, which may prevent lipid deposition in the vessel wall. The results of the study may merit new studies with longer intervention periods and a larger sample size to further define the long-term balance of benefits/risks of moderate beer intake in cardiovascular health.

## Figures and Tables

**Table 1:** Subject characteristics at baseline for phase 1 and for phase 2, at the time of starting each intervention period (*N* = 36).

	After Run-In Period	After Wash-Out Period	*p*-Value
*Anthropometric parameters*			
Sex (Men/Women)	21/15	21/15	
Weight (kg)	87.9 ± 2.3	88.2 ± 2.0	*0.92*
BMI (kg/m^2^)	30.5 ± 0.5	30.6 ± 0.5	*0.88*
Waist circumference (cm)	100.4 ± 1.7	101.5 ± 1.5	*0.63*
*Hemodynamic control*			
Systolic blood pressure (mmHg)	127.1 ± 1.8	125.6 ± 1.8	*0.57*
Diastolic blood pressure (mmHg)	75.8 ± 1.5	75.1 ± 1.3	*0.71*
Cardiac Frequency (beats/min)	65.3 ± 1.6	64.9 ± 1.4	*0.85*
*Biochemical parameters*			
Glucose (mg/dL)	88.5 ± 1.6	89.2 ± 1.7	*0.77*
Creatinine (mg/dL)	0.77 ± 0.02	0.77 ± 0.02	*0.75*
Urea (mg/dL)	14.0 ± 0.6	14.8 ± 0.5	*0.28*
AST (U/L)	16.8 ± 0.7	16.9 ± 0.7	*0.85*
GGT (U/L)	20.5 ± 1.8	22.5 ± 1.9	0.46
*Lipid parameters*			
TC (mg/dL)	188.9 ± 4.5	191.4 ± 4.5	0.67
HDLc (mg/dL)	48.3 ± 1.7	48.8 ± 1.6	0.82
Non-HDLc (mg/dL)	140.5 ± 4.1	142.6 ± 4.3	0.71
LDLc (mg/dL)	124.1 ± 3.8	125. 4 ± 3.9	0.80
VLDLc (mg/dL	16.4 ± 1.0	16.9 ± 1.0	0.58
TGL (mg/dL)	81.5 ± 4.8	85.6 ± 5.0	0.58

Baseline values after the four-week run-in and the four-week wash-out periods are expressed as mean ± SEM. Statistical analysis was performed with a Student’s *t*-test for paired samples. Statistical significance: *p* < 0.05. BMI, body mass index; AST, aspartate transaminase; GGT, gamma-glutamyltransferase; TC, total cholesterol; HDLc, high density lipoprotein cholesterol; LDLc, low density lipoprotein cholesterol; VLDLc, very low density lipoprotein cholesterol; TGL, triglycerides.

**Table 2:** Anthropometric and hemodynamic variables, biochemical parameter and hemogram profile before and after four-week dietary intervention with alcohol-free and traditional and beer.

	Alcohol-Free Beer			Traditional Beer		
	Before Intervention	After Intervention	*p*-Value	Before Intervention	After Intervention	*p*-Value
*Anthropometric parameters*						
Weight (kg)	87.7 ± 2.3	88.1 ± 2.3	*0.02*	87.7 ± 2.3	88.1 ± 2.3	*0.08*
BMI (kg/m^2^)	30.4 ± 0.5	30.5 ± 0.5	*0.01*	30.4 ± 0.5	30.4 ± 0.5	*0.11*
Waist circumference (cm)	99.7 ± 1.9	101.1 ± 1.5	*0.11*	100.5 ± 1.4	102.1 ± 1.3	*0.06*
*Hemodynamic control*						
Systolic blood pressure (mmHg)	125.4 ± 1.9	125.8 ± 1.8	*0.76*	125.3 ± 2.0	125.7 ± 2.0	*0.82*
Diastolic blood pressure (mmHg)	75.1 ± 1.6	75.2 ± 1.5	*0.93*	75.9 ± 1.5	74.9 ± 1.4	*0.36*
Cardiac Frequency (beats/min)	65.4 ± 1.7	63.9 ± 1.7	*0.25*	64.8 ± 1.5	66.0 ± 1.8	*0.25*
*Biochemical parameters*						
Glucose (mg/dL)	88.0 ± 1.6	88.8 ± 1.7	*0.43*	88.2 ± 2.0	90.1 ± 1.7	*0.04*
Creatinine (mg/dL)	0.76 ± 0.02	0.78 ± 0.13	*0.03*	0.78 ± 0.02	0.78 ± 0.02	*0.84*
Urea (mg/dL)	14.9 ± 0.6	15.0 ± 0.5	*0.75*	14.9 ± 0.6	15.6 ± 0.6	*0.21*
AST (U/L)	16.6 ± 0.7	16.3 ± 0.6	*0.49*	16.6 ± 0.6	17.2 ± 0.7	*0.14*
GGT (U/L)	21.5 ± 2.0	21.7 ± 1.9	*0.82*	20.6 ± 1.9	23.8 ± 2.0	*0.00*
*Hemogram*						
RBC (10^6^ mm)	4.3 ± 0.1	4.3 ± 0.1	*0.09*	4.4 ± 0.1	4.3 ± 0.1	*0.14*
HCT (%)	36.7 ± 0.6	36.2 ± 0.6	*0.14*	37.2 ± 0.7	36.5 ± 0.5	*0.21*
PLT (10^3^ mm^3^)	198.9 ± 5.5	204.1 ± 6.7	*0.17*	200.9 ± 6.4	205.4 ± 6.2	*0.26*
MPV (Um^3^)	8.4 ± 0.1	8.4 ± 0.1	*0.74*	8.4 ± 0.1	8.4 ± 0.1	*0.41*
WBC (10^3^ mm^3^)	5.8 ± 0.2	5.9 ± 0.2	*0.27*	5.9 ± 0.2	6.1 ± 0.2	*0.36*

Values before and after the four-week intervention period with alcohol-free and traditional beer expressed as mean ± SEM. Statistical analysis was performed with the Student’s *t*-test for paired samples. Statistical significance: *p* < 0.05. RBC, red blood cells; HCT, hematocrit; PLT, platelet; MPV, mean platelet volume; WBC, white blood cells.

**Table 3:** Evolution of weight, BMI and waist circumference for men and women during the study period.

	Beer Intervention				*p*-Value
	Week-0	Week-4	Week-8	Week-12	
*Men (N = 21)*					
Weight (kg)	94.6 ± 2.5 *[89.4–99.8]*	94.9 ± 2.6 *[89.6–100.3]*	94.3 ± 2.4 *[89.2–99.4]*	94.8 ± 2.5 *[89.6–100.1]*	*0.99*
BMI (kg/m^2^)	30.2 ± 0.5 *[29.1–31.3]*	30.4 ± 0.5 *[29.3–31.5]*	30.5 ± 0.5 *[29.3–31.6]*	30.4 ± 0.5 *[29.3–31.5]*	*0.99*
Waist-circumference (cm)	103.3 ± 2.2 *[98.7–108.0]*	103.7 ± 1.9 *[99.8–107.6]*	102.4 ± 1.6 *[99.2–105.7]*	103.9 ± 1.5 *[100.8–107.1]*	*0.88*
*Women (N = 15)*					
Weight (kg)	78.6 ± 2.2 *[72.5–84.6]*	78.8 ± 3.0 *[72.5–85.1]*	77.9 ± 3.1 *[71.3–84.5]*	78.4 ± 3.0 *[71.9–84.9]*	*0.99*
BMI (kg/m^2^)	30.8 ± 0.9 *[28.8–32.7]*	30.8 ± 0.9 *[28.8–32.8]*	30.5 ± 1.0 *[28.4–32.6]*	30.6 ± 1.0 *[28.6–32.7]*	*0.44*
Waist-circumference (cm)	96.3 ± 2.5 *[91.0–101.7]*	98.4 ± 2.3 *[93.5–103.3]*	97.4 ± 2.5 *[92.1–102.7]*	98.3 ± 1.9 *[94.2–102.4]*	*0.94*

Values are given as mean ± SEM [95% confidence interval]; *p*-values obtained by analysis of variance (ANOVA) for 1 factor. Statistical significance: *p* < 0.05. The time-interval between week-4 and week-8 refers to the wash-out period. Beer intervention includes traditional and alcohol-free beer intake. BMI, body mass index.

**Table 4:** Serum lipid levels before and after four-week dietary intervention with alcohol-free and traditional beer.

	Alcohol-Free Beer			Traditional Beer		
	Before Intervetion	After Intervention	*p*-Value	Before Intervetion	After Intervention	*p*-Value
TC (mg/dL)	189.3 ± 4.5	191.0 ± 4.7	*0.63*	189.9 ± 5.0	193.1 ± 4.5	*0.33*
HDLc (mg/dL)	47.7 ± 1.6	48.0 ± 1.7	*0.69*	48.2 ± 1.6	48.8 ± 1.5	*0.41*
Non-HDLc (mg/dL)	141.6 ± 4.0	143.0 ± 4.4	*0.65*	141.7 ± 4.5	144.2 ± 4.4	*0.39*
LDLc (mg/dL)	124.8 ± 3.8	125.6 ± 4.0	*0.78*	125.7 ± 4.3	126.1 ± 3.9	*0.88*
VLDLc (mg/dL)	16.8 ± 1.1	17.4 ± 1.2	*0.43*	16.0 ± 0.9	18.1 ± 1.4	*0.06*
TG (mg/dL)	83.5 ± 5.5	86.4 ± 6.0	*0.43*	79.5 ± 4.4	90.1 ± 6.9	*0.06*

Lipid data (mg/dL) are given as mean ± SEM. Differences for values before and after four-week intervention with alcohol-free and traditional beer were analyzed by paired Student’s *t*-test. Statistical significance: *p* < 0.05. TC, total cholesterol; HDL, high density lipoproteins; LDL, low density lipoproteins; TG, triglycerides; VLDL, very-low density lipoproteins.

**Table 5:** Serum lipid levels before and after four-week dietary intervention with alcohol-free and traditional beer in subjects with LDLc at baseline below and above 130 mg/dL.

	Alcohol-Free Beer Intervention				Traditional Beer Intervention			
	Before	After	Δ	*p*-Value	Before	After	Δ	*p*-Value
**Serum lipids-subjects with LDL < 130 mg/dL**								
CT (mg/dL)	170.5 ± 3.5	176.5 ± 4.0	+6.0	*0.14*	170.6 ± 2.5	179.1 ± 3.8	+8.6	***0.04***
HDLc (mg/dL)	46.3 ± 2.2	47.0 ± 2.2	+0.7	*0.34*	46.6 ± 1.7	48.8 ± 1.7	+2.2	***0.01***
Non-HDLc (mg/dL)	124.2 ± 3.1	129.5 ± 4.3	+5.2	*0.17*	124.9 ± 2.6	130.5 ± 3.7	+5.6	*0.12*
LDLc (mg/dL)	108.8 ± 2.9	112.4 ± 3.5	+3.6	*0.35*	109.9 ± 2.2	114.2 ± 3.4	+4.3	*0.18*
VLDLc (mg/dL)	15.4 ± 1.4	17.1 ± 1.6	+1.6	*0.08*	15.0 ± 1.1	16.3 ± 1.3	+1.3	*0.13*
TG (mg/dL)	76.8 ± 6.8	84.9 ± 8.1	+8.2	*0.08*	74.7 ± 5.3	81.2 ± 6.7	+6.6	*0.13*
**Serum lipids-subjects with LDL > 130 mg/dL**								
CT (mg/dL)	215.6 ± 3.7	211.3 ± 6.9	−4.3	*0.49*	226.1 ± 5.5	221.4 ± 4.6	−4.7	*0.22*
HDLc (mg/dL)	49.7 ± 2.5	49.3 ± 2.6	−0.3	*0.82*	50.9 ± 3.5	49.7 ± 3.0	−1.1	*0.46*
Non-HDLc (mg/dL)	165.9 ± 2.6	161.9 ± 5.7	−4.0	*0.43*	175.2 ± 3.9	171.6 ± 4.8	−3.5	*0.52*
LDLc (mg/dL)	147.2 ± 2.5	144.1 ± 5.3	−3.1	*0.51*	157.2 ± 4.8	149.9 ± 4.7	−7.3	*0.16*
VLDLc (mg/dL)	18.7 ± 1.7	17.8 ± 1.9	−0.9	*0.45*	17.9 ± 1.5	21.7 ± 3.0	+3.7	*0.22*
TG (mg/dL)	92.9 ± 8.7	88.4 ± 9.3	−4.5	*0.45*	89.2 ± 7.6	107.8 ± 14.9	+18.6	*0.22*

Serum lipid values for subjects with moderate CVD risk (LDL < 130 mg/dL) and high (LDL > 130 mg/dL) are given as mean ± SEM. Differences for values before and after four-week intervention with alcohol-free and traditional beer were analyzed by paired Student’s *t*-test. ∆ Percentage of change in plasma lipids levels after four-week intervention with alcohol-free and traditional beer. *p* ≤ 0.05 indicates statistical significance. TC, total cholesterol; HDL, high density lipoproteins; LDL, low density lipoproteins; TG, triglycerides; VLDL, very-low density lipoproteins.

**Table 6:** Effect of the intervention with alcohol-free and traditional beer on the cholesterol efflux induced by apoB-depleted serum in macrophages.

Cholesterol Efflux (%)	Alcohol-Free Beer			Traditional Beer		
	Before Intervention	After Intervention	*p*-Value	Before Intervention	After Intervention	*p*-Value
Total population (*N* = 36)	16.4 ± 0.4	16.6 ± 0.4	*0.16*	16.6 ± 0.4	17.2 ± 0.4	***0.02***
*Men (N = 21)*	16.9 ± 0.4	17.1 ± 0.5	*0.47*	16.9 ± 0.5	17.6 ± 0.5	*0.09*
*Women (N = 15)*	15.6 ± 0.7	16.0 ± 0.6	*0.21*	16.4 ± 0.7	16.8 ± 0.7	*0.45*

Cholesterol efflux values expressed as percentage and given as the mean ± SEM; *p*-values were analyzed by a Student’s *t*-test for paired samples.

**Table 7:** Effect of the intervention with alcohol-free and traditional beer on inflammatory markers.

Inflammatory Markers	Alcohol-Free Beer			Traditional Beer		
	Before Intervention	After Intervention	*p*-Value	Before Intervention	After Intervention	*p*-Value
PCR (ng/mL) TNFα (ng/mL) IL-6 (ng/mL)	3.4 ± 0.6 83.1 ± 0.96 0.03 ± 0.00	4.1 ± 0.8 2.0 ± 0.1 0.03 ± 0.01	*0.06* *0.25* *0.20*	4.8 ± 1.2 1.8 ± 0.1 0.03 ± 0.01	4.4 ± 0.7 2.0 ± 0.2 0.04 ± 0.01	*0.63* *0.06* *0.58*

Values of the inflammatory markers expressed in ng/mL and given as the mean ± SEM; *p*-values were obtained by Student’s *t*-test for paired samples. CRP, C-reactive protein; TNF-α, tumor necrosis factor alpha; IL-6, interleukin-6.

**Table 8:** Effect of beer consumption on the Framingham Risk Score (FRS).

	Alcohol-Free Beer				Traditional Beer			
	Before	After	10-Year Risk	*p*-Value	Before	After	10-Year Risk	*p*-Value
Total population (*N* = 36)	7.9 ± 0.5	7.8 ± 0.5	<1%	0.50	7.9 ± 0.5	7.8 ± 0.5	<1%	0.50
*Men (N = 21)*	7.3 ± 0.5	7.2 ± 0.5	3%	0.54	7.3 ± 0.5	7.2 ± 0.5	3%	0.54
*Women (N = 15)*	8.7 ± 1.1	8.7 ± 1.1	<1%	0.75	8.7 ± 1.1	8.7 ± 1.1	<1%	0.75

The Framingham score was calculated according to the guidelines provided in the Framingham Heart Study of NHLBI (National Heart, Lung & Blood Institute & Boston University) [29]. Values are expressed as mean ± SEM was analyzed by Student’s *t*-test for paired samples.

## Supplementary Materials

The following are available online at http://www.mdpi.com/2072-6643/10/9/1237/s1, Supplementary Table S1: Phenolic composition of the beers used in the study, Supplementary Table S2: Anthropometric and hemodynamic variables, biochemical parameter and hemogram profile before and after four-week dietary intervention with alcohol-free and traditional and beer in men (*N* = 21), Supplementary Table S3: Anthropometric and hemodynamic variables, biochemical parameter and hemogram profile before and after four-week dietary intervention with alcohol-free and traditional and beer in women (*N* = 15).
