# Supplementary material for: Moderate Beer Intake and Cardiovascular Health in Overweight Individuals
Source: Nutrients. 2018 Sep 5;10(9):1237. doi: 10.3390/nu10091237 (PMC6164820; doi:10.3390/nu10091237)
Supplement: Supplementary file 1 [file nutrients-10-01237-s001.pdf]

Supplementary Materials: The following are available online at www.mdpi.com/xxx/s1,

Supplementary Table S1.- Phenolic composition of the beers used in the study

**Phenolic compounds**	**Traditional Beer**		**Alcohol-free Beer**	
	**Men**	**Women**	**Men**	**Women**
4hydroxybenzoic acid	47.4 ± 1.0	23.7 ± 1.0	26.4 ± 1.0	13.2 ± 1.0
Caffeic acid	43.2 ± 1.0	21.6 ± 1.0	37.2 ± 1.0	18.6 ± 1.0
Catechin	94.2 ± 2.0	47.1 ± 1.0	99 ± 3.0	49.5 ± 3.0
Chlorogenic acid	6.6 ± 0.2	3.3 ± 0.1	1.4 ± 0.0	0.7 ± 0.0
Epicatechin	12.7 ± 0.2	6.4 ± 0.2	12.9 ± 0.2	6.5 ± 0.2
Ferulic acid	333 ± 6.0	166.5 ± 4.0	241.8 ± 5.0	120.9±5.0
Kaempferol-*O*-glucoside	6.2 ± 0.2	3.1 ± 0.2	4.9 ± 0.1	2.5 ± 0.1
p-Coumaric acid	141.0 ± 4.0	70.5 ± 3.0	92.4 ± 2.0	46.2 ± 2.0
Protocatechuic acid	22.4 ± 0.7	11.2 ± 0.4	13.6 ± 0.3	6.8 ± 0.3
Quercetin	9.9 ± 0.2	4.9 ± 0.2	8.5 ± 0.3	4.3 ± 0.3
Quercetin-3-*O*-glucoside	37.4 ± 1.1	18.7 ± 0.7	21.8 ± 0.4	10.9 ± 0.4
Rutin	10.1 ± 0.2	5.0 ± 0.1	7.1 ± 0.3	3.6 ± 0.3
Sinapic acid	77.1 ± 1.0	38.7 ± 1.0	54.6 ± 1.0	27.3 ± 1.0
Vanillic acid	17.0 ± 0.5	8.5 ± 0.3	7.2 ± 0.3	3.6 ± 0.3
Isoxanthohumol	331.2 ± 49.0	165.6 ± 32.0	111.6 ± 8.0	55.8 ± 8.0
8-Prenylnaringenin	19.6 ± 1.4	9.8 ± 0.9	11.4 ± 1.2	5.7 ± 1.2
Total (sum)	1209.6 ± 131.0	604.8 ± 190.0	751.8 ± 158.0	375.9 ± 157.0

Daily intake (µg). Values are given as mean±SD

Supplementary Table S2. Anthropometric and hemodynamic variables, biochemical parameter and hemogram profile before and after 4-weeks dietary intervention with alcohol-free and traditional and beer in men (N=21)

	Alcohol-free Beer			Traditional Beer		
	**Baseline**	**Δ**	***P-value***	**Baseline**	**Δ**	***P-value***
Anthropometric parameters						
Weight (Kg)	94.4±2.4	0.5±0.3	*0.06*	94.5±2.5	0.4±0.3	*0.24*
BMI (Kg/m^2)	30.2±0.5	0.1±0.1	*0.14*	30.3±0.5	0.1±0.1	*0.32*
Waist (cm)	102,9±2.2	0.9±1.1	*0.39*	102.9±1.6	0.9±0.9	*0.32*
Hemodynamic control						
Systolic blood pressure (mmHg)	127.4±1.8	-0.2±1.5	*0.90*	126.2±2.0	0.4±2.7	*0.89*
Diastolic blood pressure (mmHg)	79.0±1.5	-0.7±1.0	*0.47*	78.1±1.4	-1.9±1.4	*0.19*
Cardiac Frequency (beats/min)	64.1±2.3	-3.7±1.1	***<0.01***	62.9±2.2	1.6±0.7	***0.02***
Biochemical parameters						
Glucose (mg/dL)	89.1±2.3	1.0±1.2	*0.40*	90.7±2.5	0.4±1.1	*0.69*
Creatinine (mg/dL)	0.83±0.02	0.03±0.01	***0.03***	0.87±0.03	0.001±0.01	*0.90*
Urea (mg/dL)	15.0±0.8	0.2±0.6	*0.78*	15.7±0.9	0.4±0.9	*0.61*
AST (U/L)	17.5±0.8	-1.0±0.5	*0.08*	17.0±0.8	0.9±0.5	*0.12*
GGT (U/L)	24.7±2.4	1.3±0.5	***0.02***	24.8±2.8	4.5±0.9	***<0.01***
Hemogram						
RBC (10^6mm)	4.5±0.1	-0.04±0.04	*0.32*	4.5±0.1	-0.03±0.04	*0.46*
HCT(%)	38.4±0.6	-0.3±0.4	*0.43*	38.1±0.5	-0.03±0.04	*0.93*
PLT (10^3 mm^3)	187.9±5.9	-1.2±4.0	*0.77*	191.8±6.8	-1.6±4.0	*0.70*
MPV (Um^3)	8.4±0.1	-0.04±0.04	*0.61*	8.4±0.1	-0.01±0.09	*0.91*
WBC (10^3 mm^3)	5.9±0.2	0.2±0.2	*0.12*	6.0±0.2	0.1±0.2	*0.65*

Baseline values after the 4-week run-in and the 4-week wash-out periods are expressed as mean ± SEM. Statistical analysis was performed with a T-student for paired samples. Statistical significance: $p<0.05$. BMI=body mass index; AST=aspartate transaminase; GGT= gamma-glutamyltransferase; RBC= red blood cells; HCT= hematocrit; PLT= platelet; MPV= mean platelet volume; WBC= white blood cells.

Supplementary Table S3. Anthropometric and hemodynamic variables, biochemical parameter and hemogram profile before and after 4-weeks dietary intervention with alcohol-free and traditional and beer in women (N=15)

	Alcohol-free Beer			Traditional Beer		
	**Baseline**	**Δ**	***P-value***	**Baseline**	**Δ**	***P-value***
Anthropometric parameters						
Weight (Kg)	78.4±3.0	0.2±0.2	*0.20*	78.1±2.8	0.5±0.4	*0.21*
BMI (Kg/m^2)	30.6±1.0	0.1±0.1	*0.14*	30.6±0.9	0.2±0.1	*0.26*
Waist (cm)	95.1±3.2	2.0 ±1.3	*0.15*	97.3±2.3	2.4±1.4	*0.11*
Hemodynamic control						
Systolic blood pressure (mmHg)	122.5±3.9	1.3±2.6	*0.63*	124.0±3.9	0.4±1.9	*0.84*
Diastolic blood pressure (mmHg)	69.7±2.9	1.2±2.0	*0.56*	72.8±2.7	0.4±1.5	*0.79*
Cardiac Frequency (beats/min)	67.2±2.3	1.7±2.4	*0.50*	67.5±1.9	0.5±2.3	*0.82*
Biochemical parameters						
Glucose (mg/dL)	86.6±2.1	0.2±1.3	*0.86*	84.7±1.8	4.1±1.5	***0.02***
Creatinine (mg/dL)	0.67±0.05	0.01±0.01	*0.59*	0.67±0.01	0.01±0.01	*0.63*
Urea (mg/dL)	14.6±0.9	0.1±0.7	*0.88*	13.7±0.9	1.2±0.7	*0.13*
AST (U/L)	15.3±1.1	0.7±0.5	*0.18*	16.0±0.91	0.2±0.6	*0.75*
GGT (U/L)	17.1±3.1	-1.5±1.6	*0.36*	14.7±1.2	1.5±0.6	***0.02***
Hemogram						
RBC (10^{6}mm)	4.0±0.1	-0.1 ±0.1	*0.18*	4.2±0.2	-0.2±0.1	*0.21*
HCT(%)	34.3±0.7	-0.7±0.5	*0.22*	36.0±1.6	-1.7±1.2	*0.19*
PLT (10^3 mm^3)	214.4±9.1	14.1±6.4	***0.05***	213.7±11.7	12.9±7.0	*0.09*
MPV (Um^3)	8.3±0.2	0.1±0.1	*0.40*	8.4±0.3	-0.1±0.2	*0.35*
WBC (10^3 mm^3)	5.6±0.3	0.0±0.2	*>0.99*	5.8±0.3	0.2±0.3	*0.43*

Baseline values after the 4-week run-in and the 4-week wash-out periods are expressed as mean ± SEM. Statistical analysis was performed with a T-student for paired samples. Statistical significance: $p<0.05$. BMI=body mass index; AST=aspartate transaminase; GGT= gamma-glutamyltransferase; RBC= red blood cells; HCT= hematocrit; PLT= platelet; MPV= mean platelet volume; WBC= white blood cells.
